# Bibliographical Notices

**Published:** 1847-06

**Authors:** 


### BIBLIOGROPHICAL NOTICES.

*Charge to the Graduates of Jefferson Medical College of Philadeladelphia; delivered March* 25, 1847. By Professor Dunglison.

The well known good judgment of the author characterises this production. We quote one paragraph, which, in our estimation, contains excellent advice:

" To preserve yourselves on a level with the medical literature of the day, let me recommend you to peruse regularly, but with due caution in sifting the facts from the assumptions—the grain from the chaff—the pages of a good medical periodical. Whoever is remote from a large town has necessarily more or less difficulty in procuring the more ponderous works that issue, from time to time, from the press; but owing to the astonishing celerity of communication between every portion of this extensive continent, and between it and the old world, an acquaintance with the novelties of medical observation and reflection can be transmitted by means of the Journals with wonderful rapidity to every inquirer, no matter how distant he may be from the great centres of population."

*Address delivered before the Genesee Co. Medical Society at its annual Meeting, held at Batavia, Jan.* 12, 1847. By Moses Barrett, M. D.

We have been remiss in not before returning our acknowledgements for this excellent address. The author particularises with caustic severity some of the dishonorable and ungenerous means which unworthy members of our profession pursue to depreciate others, with the hope thereby of elevating themselves. It is to this, more than any other source, that we are to look for the causes which bring upon Medical Science want of confidence and respect with the Public. The surest way to recommend our profession to general esteem, is to manifest our own regard for it, by the strict observance of honor and courtesy, in all our relations with those who, as well as ourselves, are its representatives.

It is creditable to the Medical society of our neighboring county that they solicited a copy of this address for publication. We hope it will be extensively circulated.

---

*Dr. Blakeman's address to the candidates for degrees of Licences in the Medical Institution of Yale College, Jan.* 20, 1847.

Did our limits permit we should be glad to make extracts from this, as well as from various other addresses, made on similar occasions, which we receive. We trust the newly fledged candidates for medical practice will not forget the parting words of advice with which their teachers bid them God speed.

---

*Address delivered to the Graduating class of the Indiana Medical College, at the Public Commencement, Feb.* 18, 1847. By M. L. Knapp, M. D., Prof. of Materia Medica, &c.

With much that is sound and judicious in this address, there are some things obnoxious to criticism. The following extract, for example, we conceive is in bad taste:

"But gentlemen, under the lights of observation and experience afforded your teachers in Western practice, in the treatment of our endemic diseases, you will go forth from this school of medicine, with a pathology and therapia that will avail you in the West like a magic wand, or as the brazen serpent did Moses in the Israelitish camp. Western people have already seen the Eastern bred physician succumb to the superior skill of the graduates of this college, and the opinion that students must graduate at Laporte in order to practice successfully in the Northwest, is becoming prevalent here. And humanity exhorts us to foster and promulge it; and policy will yet bring many students annually from the East, to graduate in the Indiana Medical College, and to disperse, like you through the west, to become the conservators of the life and health of the tide of immigration that is rolling in upon us.

---

*Address to the Graduates of Geneva Medical College, Jan. 26, 1847.* By Charles Alfred Lee, M. D. Prof. of Gen Pathology and Materia Medica, &c.

As would be expected from the reputation of the author, this address is replete with elevated sentiments, embodied in a chaste and vigorous style.

---

*Braithwaite's Retrospect of Practical Medicine and Surgery.* Part the Fourteenth. New-York, Daniel Adee, 107 Fulton-St., 1847, pp. 357. Price 75 cents, $1,50 per annum.

*The Half-Yearly Abstract of the Medical Sciences.* Being a practical and analytical digest of the contents of the principal British and Continental Medical works published during the preceding six months; together with a series of critical reports on the progress of Medicine, and the Natural Sciences, during the same period. Edited by W. H. Ranking, M. D. Cantab. Vol. 11, No. 2, 1847. Philadelphia, Lindsay and Blakeston; 75 cts per number, $1,50 per annum; pp. 411.

The popularity and merits of these two works continue unabated.

